# Caloric restriction exacerbates renal post-ischemic injury and fibrosis by modulating mTORC1 signaling and autophagy

**DOI:** 10.1016/j.redox.2025.103500

**Published:** 2025-01-16

**Authors:** Lang Shi, Hongchu Zha, Juan Zhao, Haiqian An, Hua Huang, Yao Xia, Ziyu Yan, Zhixia Song, Jiefu Zhu

**Affiliations:** a Department of Nephrology, The First Hospital of Lanzhou University, Lanzhou, 730000, China; b The First Clinical Medical College, Lanzhou University, Lanzhou, 730000, China; c Department of Nephrology, The First Clinical Medical College of Three Gorges University, Center People's Hospital of Yichang, Yichang, 443000, China; d Department of Laboratory Medicine, The First Hospital of Lanzhou University, Lanzhou, 730000, China; e Department of Nephrology, The People's Hospital of Longhua, Shenzhen, 518109, China; f Department of Organ Transplantation, Renmin Hospital of Wuhan University, Wuhan, 430060, China

**Keywords:** Acute kidney injury, Caloric restriction, mTORC1, Ischemia-reperfusion injury, Autophagy

## Abstract

**Objective:**

This study investigates the effects of caloric restriction (CR) on renal injury and fibrosis following ischemia-reperfusion injury (IRI), with a focus on the roles of the mechanistic/mammalian target of rapamycin complex 1 (mTORC1) signaling and autophagy.

**Methods:**

A mouse model of unilateral IRI with or without CR was used. Renal function was assessed through serum creatinine and blood urea nitrogen levels, while histological analysis and molecular assays evaluated tubular injury, fibrosis, mTORC1 signaling, and autophagy activation. Inducible renal tubule-specific Atg7 knockout mice and autophagy inhibitor 3-MA were used to elucidate autophagy's role in renal outcomes.

**Results:**

CR exacerbated renal dysfunction, tubular injury, and fibrosis in IRI mice, associated with suppressed mTORC1 signaling and enhanced autophagy. Rapamycin, an mTORC1 inhibitor, mimicked the effects of CR, further supporting the involvement of mTORC1-autophagy pathway. Tubule-specific Atg7 knockout and autophagy inhibitor 3-MA mitigated these effects, indicating a central role for autophagy in CR-induced renal damage. Glucose supplementation, but not branched-chain amino acids (BCAAs), alleviated CR-induced renal fibrosis and dysfunction by restoring mTORC1 activation. Finally, we identified leucyl-tRNA synthetase 1 (LARS1) as a key mediator of nutrient sensing and mTORC1 activation, demonstrating its glucose dependency under CR conditions.

**Conclusion:**

Our study provides novel insights into the interplay between nutrient metabolism, mTORC1 signaling, and autophagy in IRI-induced renal damages, offering potential therapeutic targets for mitigating CR-associated complications after renal IRI.

## 1 Introduction

Acute kidney injury (AKI) is a significant clinical problem with high morbidity and mortality, often resulting in permanent kidney damage and progression to chronic kidney disease (CKD) [1]. Ischemia-reperfusion injury (IRI) is a common cause of AKI, leading to oxidative stress, inflammation, and tubular damage [[2], [3], [4]]. Even after severe injury, the kidneys have the potential to recover. Renal tubular epithelial cells have the ability to dedifferentiate, proliferate, and redifferentiate to repair damaged nephrons. However, maladaptive tubular repair can result in renal dysfunction and structural damage, leading to CKD, a process influenced by the severity of the injury and the capacity for recovery.

Caloric restriction (CR) is a well-established intervention known to extend lifespan and confer protection against various metabolic diseases in multiple organisms [[5], [6], [7]]. CR exerts its effects by modulating key metabolic pathways, including the inhibition of the mechanistic target of rapamycin (mTOR) and the activation of autophagy, a cellular process that degrades damaged organelles and proteins [8,9]. While CR has been shown to be beneficial in metabolic health and weight management, recent studies have revealed its adverse effects on wound healing, infection [10],and certain types of tissue regeneration [11]. Currently, the impact of caloric restriction on tissue regeneration following AKI remains unclear.

In response to nutrient availability, the mTORC1 pathway plays a central role in regulating cellular growth, metabolism, and survival [12,13]. The activation of mTOR in renal tubular cells after AKI is essential for tubule repair [14,15], although several reports suggest that mTOR inhibition can also have protective effects [16]. Therefore, the role of mTOR in AKI is complex and may be context-dependent. Additionally, mTOR influences autophagy, a process generally considered crucial in the context of chronic kidney disease [17,18]. Although finely regulated autophagy is essential for kidney repair and regeneration, recent studies suggest that excessive or dysregulated autophagy could exacerbate tissue injury under fibrotic conditions [19,20].

In this study, we aimed to explore the effects of CR on renal injury and fibrosis following IRI and to uncover the underlying mechanisms. Surprisingly, we found that CR not only failed to mitigate the progression of AKI to CKD but also exacerbated renal dysfunction and fibrosis during this process. Through omics analysis, we focused on the roles of mTORC1 signaling and autophagy to uncover the reasons behind this unexpected outcome. Our findings shed new light on the intricate interplay between metabolism, autophagy, and renal injury, offering valuable insights into the therapeutic implications of CR and mTORC1 modulation in kidney disease.

## 2 Materials and methods

### 2.1 Animal models and treatments

All animal experiments were approved by the Ethics Committee of Three Gorges University (202205010T2). Male C57BL/6 mice (8–10 weeks old) were purchased from the Experimental Animal Center of China Three Gorges University (Yichang, China). Autophagy reporter mice (CAG-RFP-GFP-LC3) were purchased from the Jackson Laboratory (stock No: 027139). Following the experiment, CAG-RFP-GFP-LC3 mice were perfused with 4 % paraformaldehyde. The extracted kidneys were post-fixed overnight in the same fixative, equilibrated in 30 % sucrose, and embedded in Tissue-Tek® O.C.T. compound. Cryosections were then prepared for observation and image acquisition under a fluorescent confocal microscope. For the IRI model, mice were anesthetized with isoflurane, and the left kidney was subjected to ischemia by clamping the renal pedicle for 28 min, followed by reperfusion. The right kidney was removed via uninephrectomy at day 13 post-IRI, and mice were sacrificed on day 14 [4,21]. On the fourth day post-surgery, mice in the calorie restriction group were fed at 70 % of the normal intake, while control and sham-operated groups had ad libitum feeding. All groups had free access to water. Mice were monitored daily, and their condition was recorded. On day 13 of reperfusion, a right nephrectomy was performed, and 24 h later, blood was collected from the left renal artery.

For autophagy inhibition studies, mice were treated with 3-MA (15 mg/kg, intraperitoneally) daily starting on day 4 post-IRI. For mTORC1 inhibition, mice received rapamycin (Rap) (2.5 mg/kg/day, intraperitoneally) starting on day 4 post-IRI. In the study on glucose, BCAA, aspartate, or asparagine supplementation, we administered 0.14 g/ml of glucose or the respective amino acids via drinking water.

### 2.2 Gene delivery in vivo

In the animal experiments, adenoviruses(Ad) carrying ribosomal protein S6 kinase, polypeptide 1(Rps6kb1) or its nonsense sequence (negative control, NC) (Sangon Biotech, Shanghai, China) were introduced into the mouse kidneys immediately following renal ischemia, as described previously [22]. Briefly, 100 μL of adenovirus (10^8 transduction units/μL) was administered into the kidneys of anesthetized mice using a 30 G syringe. The needle was inserted at the lower pole of the kidney and advanced towards the upper pole, with the adenoviruses solution being infused slowly while the needle was gradually withdrawn.

### 2.3 Generation of inducible renal tubule-specific Atg7 knockout mice

Male C57BL/6J mice were housed in the animal facilities at China Three Gorges University and were treated humanely according to guidelines of the Institutional Animal Use and Care Committee with free access to water and food. An inducible and conditional system was used to knockout Atg7 in the renal tubule of adult mice. Three transgenic mouse lines were cross-bred: Pax8-reverse tetracycline-dependent transactivator (Pax8-rtTA) mice(Jackson lab, stock No: 007176), tetO-Cre mice (Jackson lab, stock No: 006234) and Atg7 flox/flox mice(Jackson lab, stock No:034429). Tail DNA from all mice was genotyped by PCR analysis. Male mice aged 6–8 weeks were randomly assigned into different groups, with at least six mice per group: the non-postconditioning (Non CR) group with normal diet, calorie restriction group (CR). In the IRI model, mice were anesthetized with isoflurane, and ischemia was induced in the left kidney by clamping the renal pedicle for 28 min, followed by reperfusion. Starting on the fourth day post-unilateral IRI surgery, doxycycline (Sigma-Aldrich, D9891) was dissolved in drinking water containing 5 % sucrose and administered to the mice at a concentration of 2 mg/ml for one week to induce Atg7 deletion in renal tubules. Genotyping of genomic DNA extracted from kidney tissues was performed by PCR using primer Atg7s (5′- CTTCTGCAAGGCCCACTAAC-3′) and primer Atg7 96–121c (5′- CCCGGAGAAGTCTGAGTCTG-3′). The calorie restriction group was fed a limited diet, with daily food intake at 70 % of the normal level, while the control group was allowed ad libitum feeding after ischemia surgery, and the sham-operated group was also fed ad libitum. The condition of the mice was observed daily during restricted feeding, and records were kept. On day 13 of reperfusion, a right nephrectomy was performed, and 24 h later, the mice were anesthetized for blood collection from the left renal artery.

### 2.4 Renal function and histological analysis

Serum creatinine (CRE) and blood urea nitrogen (BUN) levels were measured by the central laboratory of the Center People's Hospital of Yichang (Roche Diagnostics GmbH, Penzberg, and Mannheim, Germany). Kidney tissues were fixed in 10 % neutral-buffered formalin, embedded in paraffin, and sectioned at 4 μm for histological analysis. Sections were stained with hematoxylin and eosin (H&E) for general histology, Masson's trichrome for fibrosis, and Sirius Red for collagen deposition. Tubular injury was scored in a blinded fashion based on the percentage of affected tubules in randomly selected fields [23]. Interstitial fibrosis was quantified as the percentage of fibrotic area relative to the total area [24].

### 2.5 Immunohistochemistry and immunofluorescence

Paraffin-embedded kidney sections were deparaffinized, rehydrated, and subjected to antigen retrieval in citrate buffer (pH 6.0). Sections were blocked with 5 % bovine serum albumin and incubated overnight at 4 °C with primary antibodies. Sections were incubated with HRP/AP-conjugated secondary antibodies and developed with DAB substrate or ImmPACT Vector Red Substrate Kit. For immunofluorescence, sections were incubated with fluorophore-conjugated secondary antibodies, including goat anti-mouse IgG conjugated with Alexa Fluor 488 or 647, or goat anti-rabbit IgG labeled with Alexa Fluor 405 (Jackson ImmunoResearch). Images were captured using a Nikon Eclipse fluorescence microscope and thePANNORAMIC, MIDI digital slide scanner (manufacturer: 3DHISTECH). Primary antibody used Immunohistochemistry and immunofluorescence including KIM1(R&D system, AF1817), LC3B(novusbio, NB100-2220), PDGFRB(ABclonal, A19531), α-SMA(Cell Signaling, 19245), FGF2(ABclonal,A11488), pS6K (ABclonal,A4898; Cell Signaling #9234), Ki67(Cell Signaling,9129T), ATG7(proteintech, 10088-2-AP), LARS1 (proteintech 21146-1-AP) Slc7a8(Thermo Fisher Scientific,ANT-108-200UL), Slc7a5(Thermo Fisher Scientific, PA5-115916), mTORC1 (SANTA CRUZ, sc-517464).Fibrosis was measured by randomly selecting 10 fields of view for each mouse kidney under a 200x magnification and then calculating the percentage of fibrotic area (collagen). The ImageJ tool was used to calculate the collagen volume fraction, that is, the percentage of the blue area (collagen) to the total area of each field.

### 2.6 Western blot analysis

Kidney tissues or cultured renal tubular cells were lysed in RIPA buffer containing protease and phosphatase inhibitors. Protein concentrations were determined using a BCA protein assay kit (Beyotime). Equal amounts of protein (30–50 μg) were separated by SDS-PAGE and transferred to PVDF membranes. Membranes were blocked with 5 % BSA and incubated overnight at 4 °C with primary antibodies against pS6K1 (Cell Signaling Technology, Thr389, 9205), S6K1 (Cell Signaling Technology, 9202), LARS1 (Bethyl Laboratories and proteintech 21146-1-AP), ULK1 (Cell Signaling Technology,8054T), *p*-ULK1 (Ser757) (Cell Signaling Technology, 6888), p62 (proteintech,18420-1-AP), FGF2 (ABclonal,A11488), mTORC1 (SANTA CRUZ, sc-517464), *p*-mTOR (ABclonal, AP0115), CTGT (ABclonal, A11067), PDGFRB (ABclonal, A19531), α-SAM (Cell Signaling, 19245), α-Tububin (Proteintech, 11224-1-AP), and GAPDH (Servicebio,GB15004-100). After washing, membranes were incubated with HRP-conjugated secondary antibodies and developed using an ECL detection system. Densitometric analysis was performed using ImageJ software.

### 2.7 Metabolomic analysis

Metabolites were extracted by homogenizing 60 mg of tissue with water and a methanol-acetonitrile solution containing internal standards, followed by protein precipitation at −20 °C and centrifugation at 14,000 rcf for 20 min at 4 °C. Chromatographic separation was performed on an Agilent 1290 UHPLC system using a gradient of ammonium acetate and acetonitrile solutions. Mass spectrometric analysis was conducted on a 6500/5500 QTRAP system with electrospray ionization in positive mode, employing multiple reaction monitoring (MRM) for quantification. Data were analyzed using MultiQuant 3.0.2, with internal standards for retention time correction and normalization.

### 2.8 Cell culture and treatment

The mouse proximal tubular cells (mPTCs) were originally obtained from Sciencell Research Laboratories and cultured in DMEM medium supplemented with 10 % fetal bovine serum and growth factors. Small interference RNAs (siRNAs) were synthesized by Sangon Biotech (Shanghai) Co., Ltd. Small interfering RNAs were transfected by Lipofectamine RNAiMAX reagent (Thermo Fisher Scientific; 13778030), according to the manufacturer's protocol. A non-silencing siRNA oligonucleotide that does not recognize any known homolog of mammalian genes was used as a negative control.

### 2.9 Glucose (Glu) or branched-chain amino acids (BCAAs) starvation and stimulation protocol

For glucose deprivation, cells were washed twice with glucose-free DMEM and then incubated in glucose-free DMEM. Stimulation was performed by adding DMEM containing 4.5 mg/ml glucose for 5–30 min. Each gram of BCAAs (Solarbio) supplement contains 0.2 g of isoleucine, 0.4 g of leucine, and 0.2 g of valine. For BCAAs starvation, cells were washed and incubated in BCAAs-free DMEM for 50 min, followed by stimulation with BCAAs-free DMEM supplemented with 800 μM BCAAs for 10 min.

### 2.10 Co-immunoprecipitation (Co-IP)

Renal tubular cells were lysed in IP lysis buffer (Beyotime) containing protease and phosphatase inhibitors. Cell lysates (500 μg of protein) were pre-cleared with Protein A/G agarose beads and incubated with anti-Flag or anti-ULK1 antibodies overnight at 4 °C. Immune complexes were captured with Protein A/G beads, washed, and eluted in SDS sample buffer. Co-immunoprecipitated proteins were analyzed by Western blotting as described above.

### 2.11 RNA sequencing and Gene Ontology pathway analyses

Total RNA extraction from renal cortex was performed with Trizol reagent (Invitrogen, USA), according to the manufacturer's instructions. The RNA concentration was calculated by a NanoDrop spectrophotometer. RNA sequencing analysis was performed by BGI (Beijing Genomics Institute) (Shenzhen, China). The differentially expressed genes were analyzed using DESeq2. The functional annotation tool at https://geneontology.org was used to conduct Gene Ontology (GO) pathway analysis. Additionally, the DAVID 6.8 functional annotation tool (https://david.ncifcrf.gov) was employed to perform KEGG pathway analysis, focusing on the identification of the top 20 enriched pathways.

### 2.12 Statistical analysis

All data are presented as mean ± SEM. Statistical significance was determined using one-way ANOVA followed by Tukey's post hoc test or two-way ANOVA as appropriate. P < 0.05 was considered statistically significant. Statistical analyses were performed using GraphPad Prism version 8.0.

## 3 Results

### 3.1 CR exacerbates renal injury and fibrosis following IRI

**Fig. 1:**
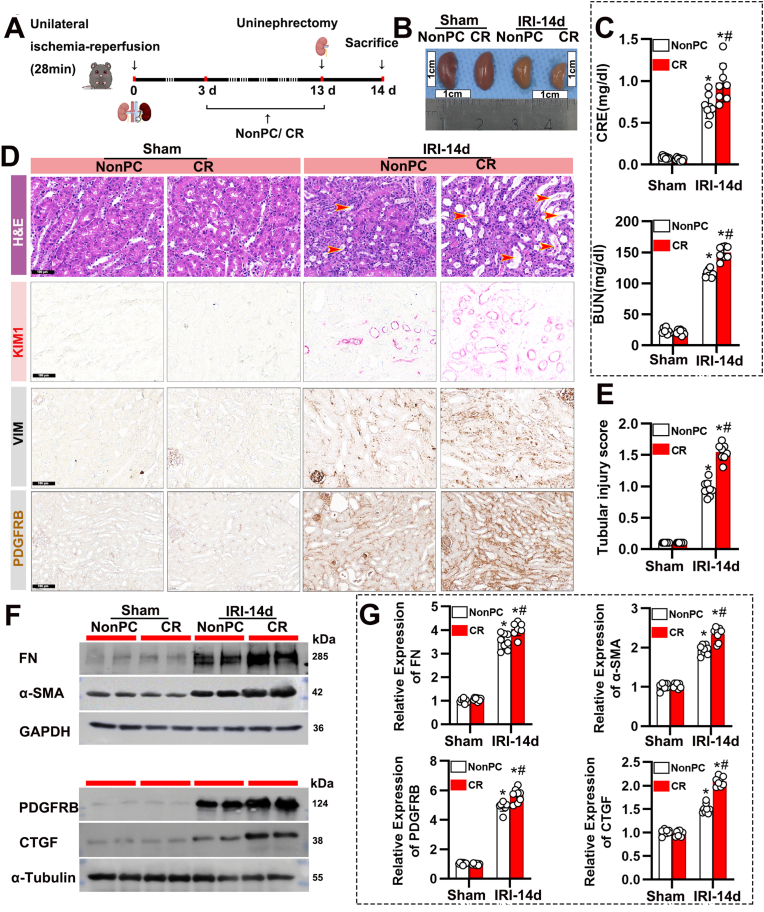
CR exacerbates renal function impairment and fibrosis induced by IRI. (A) Schematic representation of the experimental design. Mice underwent unilateral ischemia-reperfusion injury (28 min) followed by CR or NonPC for 14 days. Uninephrectomy was performed on day 13, and mice were sacrificed on day 14 for analysis. (B) Gross morphology of the kidneys from sham-operated and IRI-14d mice under NonPC and CR conditions, showing differences in kidney size and appearance. Scale bars: 1 cm. (C) CRE and BUN levels in sham and IRI-14d mice under NonPC and CR conditions. Data are presented as mean ± SEM. n = 8 per group. ∗P < 0.05 versus Sham; #P < 0.05 versus NonPC. (D) Representative images of kidney sections stained with H&E, KIM1, VIM, and PDGFRB in sham and IRI-14d mice under NonPC and CR conditions. Scale bars: 50 μm. Red arrows in the H&E panels indicate areas of tubular injury. (E) Quantification of tubular injury score in sham and IRI-14d mice under NonPC and CR conditions. Data are presented as mean ± SEM. n = 8 per group. ∗P < 0.05 versus Sham; #P < 0.05 versus NonPC. (F) Western blot analysis of fibronectin (FN), α-SMA, PDGFRB, and CTGF in kidney tissues from sham and IRI-14d mice under NonPC and CR conditions. (G) Quantification of protein expression levels of FN, α-SMA, PDGFRB, and CTGF from Western blots. Data are presented as mean ± SEM. n = 8 per group. ∗P < 0.05 versus Sham; #P < 0.05 versus NonPC.

We first assessed the impact of CR on renal injury following unilateral IRI (Fig. 1A). Mice subjected to CR after IRI (IRI-14d + CR) exhibited significantly higher levels of serum CRE and BUN compared to those in the Non-CR group, indicating exacerbated renal dysfunction (Fig. 1B and C). Immunohistochemical analysis showed elevated expression of markers associated with fibrosis and injury, including KIM1, PDGFRB, and α-SMA (Fig. 1D). Histological analysis further revealed increased tubular injury in the CR group, with elevated tubular injury scores (Fig. 1E). Additionally, we confirmed via immunoblot that FN, α-SMA, PDGFRB, and CTGF were significantly upregulated in the post-surgery CR-treated group (Fig. 1F and G). These findings suggest that CR worsens renal outcomes following IRI.

### 3.2 CR influences mTORC1 signaling and autophagy activation in the injured kidney

**Fig. 2:**
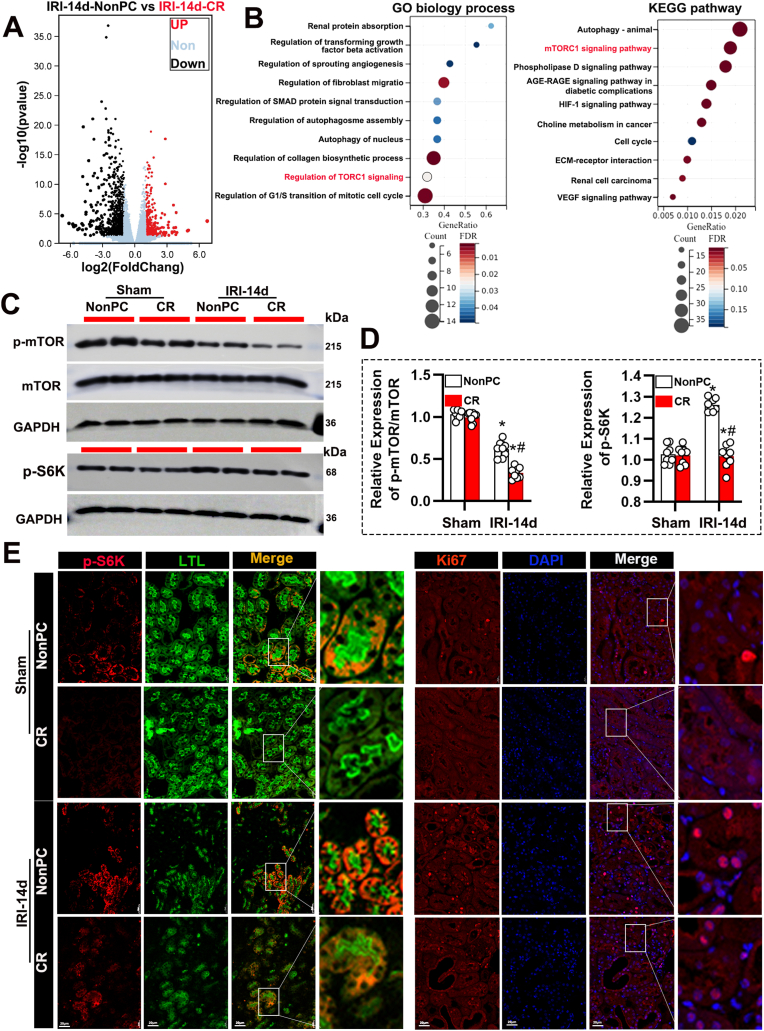
CR affects mTORC1 signaling in the kidney following IRI. (A) Volcano plot showing differentially expressed genes in the kidneys of IRI-14d mice under NonPC versus CR conditions. Genes upregulated in CR are indicated in red, and downregulated genes are indicated in black. (B) Pathway enrichment analysis of the differentially expressed genes. (C) Western blot analysis of *p*-mTOR, total mTOR, and pS6k in kidney tissues from sham and IRI-14d mice under NonPC and CR conditions. GAPDH was used as a loading control. (D) Quantification of *p*-mTOR/mTOR and pS6K expression levels from Western blots. Data are presented as mean ± SEM. n = 8 per group. ∗P < 0.05 versus Sham; #P < 0.05 versus NonPC. (E) Representative immunofluorescence images of pS6k (red) and Lotus tetragonolobus lectin (LTL, green) staining, and Ki67 (red) and DAPI (blue) staining in kidney tissues from sham and IRI-14d mice under NonPC and CR conditions. Scale bars: 20 μm. Insets show higher magnification images of the boxed regions.

Subsequently, to elucidate the molecular mechanisms underlying the deleterious effects of CR, we performed transcriptomic analysis and identified a set of differentially expressed genes (Supplemental Datasets 1). Enrichment analysis of these genes indicated that CR predominantly affects the mTORC1 signaling pathway, autophagy, and fibrosis within the kidneys (Fig. 2A and B). Immunoblot analysis revealed that CR suppressed mTORC1 signaling, as indicated by decreased phosphorylation of mTORC1 and its downstream target pS6K in CR-treated IRI mice (Fig. 2C and D). Consistent with this, immunofluorescence analysis demonstrated reduced pS6k expression in proximal tubules of CR-treated mice, along with lower Ki67 positive tubular cells, indicating decreased cell proliferation (Fig. 2E).

To further investigate the inhibitory effect of CR on the mTORC1 signaling pathway, we employed renal injection of adenoviruses to overexpress S6K (SFig. 1A). Mice subjected to unilateral IRI (28 min) were injected of adenoviruses encoding either a Ad-NC or Ad-S6K and treated with unilateral nephrectomy on day 13. The results indicated that overexpression of pS6K significantly improved renal function (SFig. 1B). Immunoblot analysis confirmed a marked increase in S6K expression in the kidneys of mice treated with Ad-S6K (SFig. 1C and D). Histological examination revealed reduced tubular damage and interstitial fibrosis in the kidneys of mice overexpressing S6K (SFig. 1E). Quantitative analysis of tubular damage scores and interstitial fibrosis percentage further supported these findings (SFig. 1F and G). These results suggest that overexpression of S6K activates the mTORC1 pathway and alleviates CR-induced renal fibrosis and dysfunction following IRI.

**Fig. 3:**
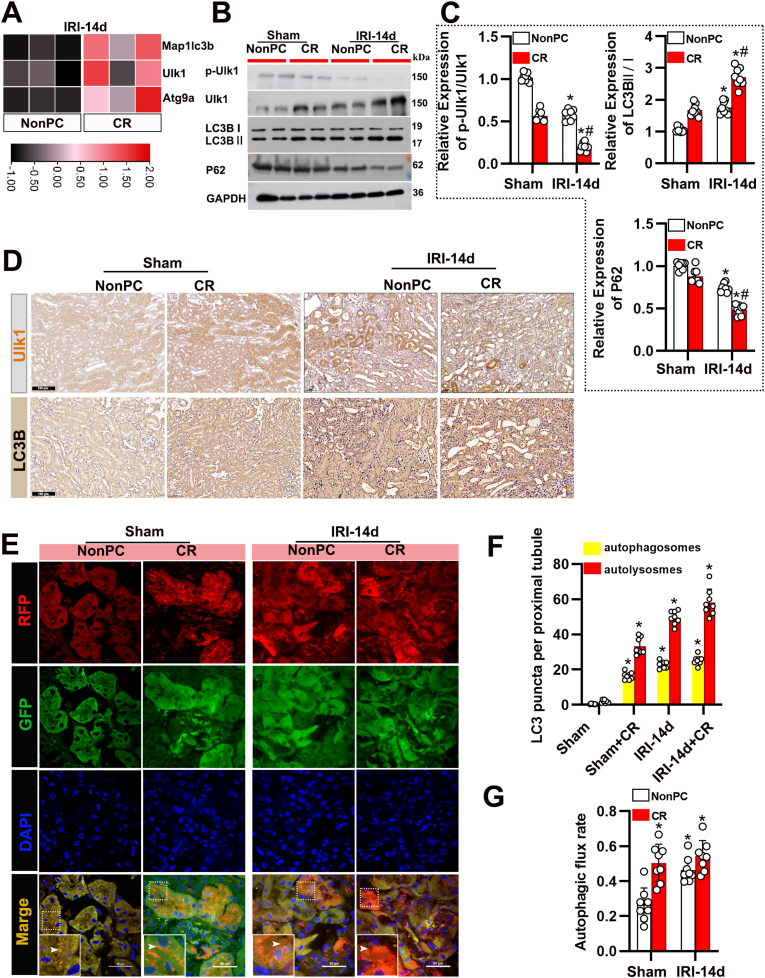
CR exacerbates autophagy activation following IRI. (A) Heatmap showing the expression levels of key autophagy-related genes (Map1lc3b, Ulk1, Atg9a) in kidneys from IRI-14d mice under NonPC and CR conditions. (B) Western blot analysis of *p*-Ulk1, total Ulk1, LC3B, and P62 in kidney tissues from sham and IRI-14d mice under NonPC and CR conditions. GAPDH was used as a loading control. (C) Quantification of *p*-Ulk1/Ulk1 and P62 expression levels from Western blots. Data are presented as mean ± SEM. n = 8 per group. ∗P < 0.05 versus Sham; #P < 0.05 versus NonPC. (D) Representative immunohistochemical images showing Ulk1 and LC3B staining in kidney tissues from sham and IRI-14d mice under NonPC and CR conditions. Scale bars: 100 μm. (E) Representative immunofluorescence images of autophagosomes (RFP, red) and autolysosomes (GFP, green) in kidney tissues from sham and IRI-14d mice under NonPC and CR conditions. Nuclei are stained with DAPI (blue). Insets show higher magnification images of the boxed regions. Scale bars: 50 μm. (F) Quantification of LC3 puncta (autophagosomes and autolysosomes) per proximal tubule in the different groups. Data are presented as mean ± SEM. n = 8 per group. ∗P < 0.05 versus Sham. (G) Autophagic flux rate quantified based on the ratio of autolysosomes to autophagosomes in the different groups. Data are presented as mean ± SEM. n = 8 per group. ∗P < 0.05 versus Sham.

We also found that CR significantly enhanced autophagy activation, as indicated by the transcriptomic data showing increased expression of autophagy-related genes (Map1lc3b, Ulk1, and Atg9a) in renal tissue (Fig. 3A). Immunoblot analysis further confirmed this, demonstrating an increase in LC3 II/I levels and a decrease in p62 levels (Fig. 3B and C). Immunohistochemical staining also corroborated these findings, showing more pronounced LC3B and ULK1-positive areas in the kidneys of CR-treated mice (Fig. 3D). To examine the location, dynamics, and flux of autophagy, we employed autophagy reporter mice expressing a tandem RFP-GFP-LC3 fusion protein. This fusion protein emits both GFP and RFP fluorescence in autophagosomes under neutral pH conditions, but the GFP signal is lost as autophagosomes mature into autolysosomes in an acidic environment [20,25]. As shown in Fig. 3E, sham-operated control mice exhibited low levels of autophagy in renal tubules, with minimal GFP-LC3 and RFP-LC3 puncta. However, following unilateral renal IRI, there was a marked increase in the formation of autophagosomes (yellow puncta representing overlapping GFP-LC3 and RFP-LC3) and autolysosomes (red-only RFP-LC3 puncta) in renal tubules at all CR treatment time points (Fig. 3E). Furthermore, autophagic flux was significantly elevated in CR-treated mice (Fig. 3F and G). These results indicate that CR inhibits the mTORC1 signaling pathway, enhances autophagy, and contributes to the exacerbation of renal injury.

### 3.3 Rapamycin mimics the effects of CR on renal injury and autophagy activation

**Fig. 4:**
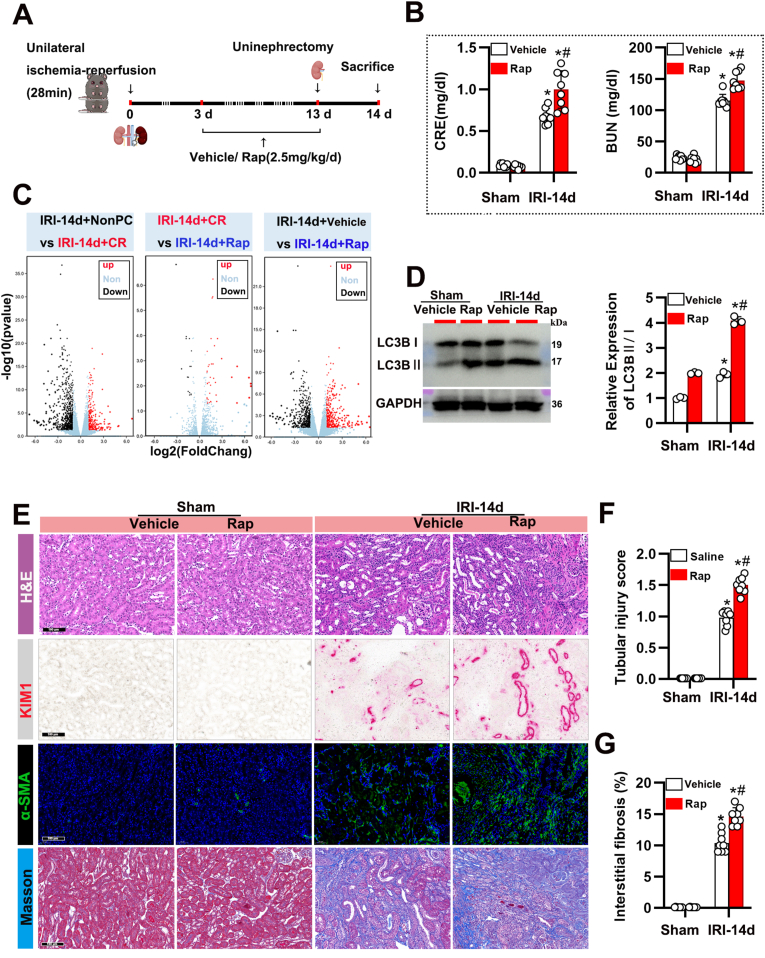
Rapamycin mimics the effects of CR on renal injury following IRI. (A) Schematic of the experimental design. Mice underwent unilateral IRI (28 min) followed by uninephrectomy and treatment with either saline or Rap (2.5 mg/kg/day) for 14 days. (B) CRE and BUN levels in sham and IRI-14d mice treated with saline or Rap. Data are presented as mean ± SEM. n = 8 per group. ∗P < 0.05 versus Sham; #P < 0.05 versus Saline. (C) Volcano plots showing differentially expressed genes in the kidneys of IRI-14d mice under the indicated conditions. Upregulated genes are shown in red, and downregulated genes are shown in black. (D) Western blot analysis of LC3B in kidney tissues from sham and IRI-14d mice treated with saline or Rap. GAPDH was used as a loading control. Quantification of LC3BⅡ/Ι expression levels is shown to the right. Data are presented as mean ± SEM. n = 8 per group. ∗P < 0.05 versus Sham; #P < 0.05 versus Saline. (E) Representative images of kidney sections stained with H&E, KIM1, α-SMA, and Masson's trichrome in sham and IRI-14d mice treated with saline or rapamycin. Scale bars: 100 μm. (F) Quantification of tubular injury score in the different groups. Data are presented as mean ± SEM. n = 8 per group. ∗P < 0.05 versus Sham; #P < 0.05 versus Saline. (G) Quantification of interstitial fibrosis percentage in the different groups, as assessed by Masson's trichrome staining. Data are presented as mean ± SEM. n = 8 per group. ∗P < 0.05 versus Sham; #P < 0.05 versus Saline.

Given that rapamycin is a specific inhibitor of mTOR, we examined whether it could mimic the effects of CR following IRI (Fig. 4A). Mice treated with rapamycin after IRI exhibited elevated serum CRE and BUN levels (Fig. 4B). The transcriptomic profiles in the middle panel (IRI-14d + CR vs IRI-14d + Rap) indicate substantial overlap between CR and rapamycin treatment, suggesting that rapamycin might replicate the effects of CR in the context of IRI through shared molecular pathways (Fig. 4C, Supplemental Datasets 2 and 3). Immunoblot and immunohistochemical analyses confirmed that rapamycin enhanced autophagy activation, as indicated by increased LC3 II/I levels and decreased p62 expression (Fig. 4D). Additionally, mice treated with rapamycin after IRI showed increased renal injury and fibrosis (Fig. 4E), elevated tubular injury scores(Fig. 4F), and increased interstitial fibrosis (Fig. 4G). These findings support the notion that CR's effects on renal injury are mediated, at least in part, through mTORC1 inhibition and enhanced autophagy.

### 3.4 Both Atg7 deficiency and autophagy inhibition through 3-MA can mitigate the detrimental effects of CR

**Fig. 5:**
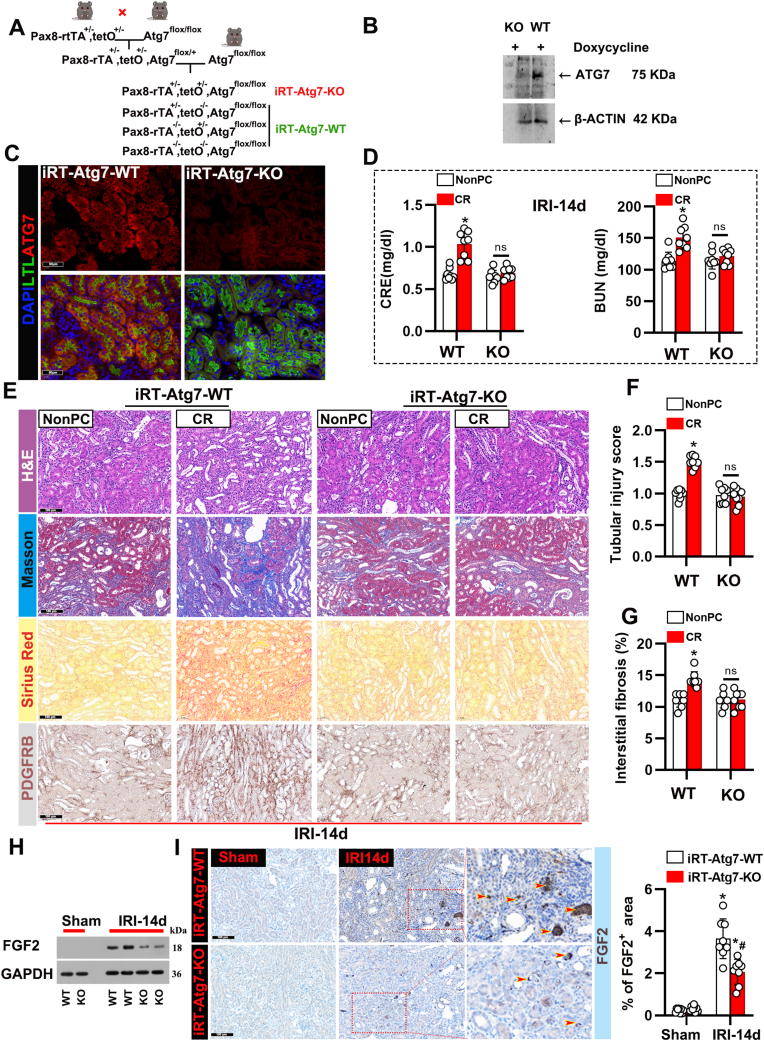
The tubule autophagy defect in iRT-atg7-KO mice inhibits the effect of CR on kidney fibrosis following ischemia. (A) Breeding strategy for generating inducible, renal tubule-specific Atg7 knockout mice. (B) At the specified time points after treatment with doxycycline, proteins were extracted from the iPT-Atg7-WT and iRT-atg7-KO kidneys, and ATG7 expression was detected by Western blot. (C) Representative immunofluorescence images of ATG7 (red) and Lotus tetragonolobus lectin (LTL, green) staining, along with DAPI (blue) staining, in renal tissue of iPT-Atg7-KO and WT mice. (D) Serum CRE and BUN levels of iPT-Atg7-KO or WT mice treated with IRI-14d under NonPC and CR conditions. Data are presented as mean ± SEM. n = 8 per group. ∗P < 0.05 versus NonPC; ns, not significant. (E) Representative images of kidney sections stained with H&E, Masson, Sirius Red and PDGFRB's trichrome from iPT-Atg7-KO and WT mice under NonPC and CR conditions. Scale bars: 100 μm. (F) Quantification of tubular injury score in the different groups. Data are presented as mean ± SEM. n = 8 per group. ∗P < 0.05 versus NonPC; ns, not significant. (G) Quantification of interstitial fibrosis percentage in the different groups, as assessed by Masson's trichrome staining. Data are presented as mean ± SEM. n = 8 per group. ∗P < 0.05 versus NonPC; ns, not significant. (H) Western blot analysis of FGF2 in kidney tissues from sham and IRI-14d mice under NonPC and CR conditions. GAPDH was used as a loading control. (I) FGF2 IHC. Scale bar: 100 μm. The right bar graph shows the quantitative analysis of the FGF2 positive staining area. Data are presented as mean ± SEM. n = 8 per group. ∗P < 0.05 versus Sham; #P < 0.05 versus iPT-Atg7-WT.

Atg7 is critical for the autophagy process, particularly in facilitating conjugation events [26]. To elucidate the role of tubular autophagy and CR in kidney repair after IRI, we generated an inducible, renal tubule-specific Atg7 knockout mouse model by breeding Pax8-rtTA^±^/tetO^±^ mice [27,28] with Atg7^flox/flox^ mice to produce iRT-atg7-KO mice and wild type (iRT-Atg7- WT) littermates (Fig. 5A). The expression of ATG7 was deleted in the kidneys of doxycycline-induced iRT-Atg7-KO mice (Fig. 5B). Immunofluorescence co-localization confirmed the loss of Atg7 in the proximal tubules of iPT-Atg7-KO mice (Fig. 5C). In contrast to wild-type (WT) mice, Atg7-deficient mice exhibited significantly reduced renal injury and fibrosis after CR, as shown by lower serum CRE and BUN levels (Fig. 5D). Histological analysis demonstrated that Atg7 deletion in CR-treated IRI mice resulted in reduced tubular injury and interstitial fibrosis, evidenced by lower injury scores and reduced fibrosis (Fig. 5E–G). In addition, mice subjected to unilateral IRI (28 min) were treated with either saline or 3-MA, with or without CR, for 14 days (SFig. 2A). Results indicated that under CR conditions, 3-MA treatment significantly reduced serum CRE and BUN levels (SFig. 2B). Immunoblot analysis demonstrated that 3-MA effectively inhibited excessive autophagy activation (SFig. 2C). Additionally, tubular injury scores further confirmed that 3-MA treatment alleviated tubular damage (SFig. 2D). Histological examination of kidney tissues from the 3-MA treatment group, using H&E, KIM1, Masson's trichrome, and LC3B staining, showed a significant reduction in interstitial fibrosis and autophagy activation (SFig. 2E and F). These results suggest that autophagy plays a critical role in mediating CR-induced renal injury. Furthermore, previous studies have demonstrated that following AKI, fibrotic tubular cells produce FGF2 via autophagy, which contributes to fibroblast activation and kidney fibrosis [29]. Immunoblot analysis showed a significant increase in FGF2 levels in WT kidneys two weeks after unilateral ischemic AKI, whereas this increase was substantially lower in iRT-Atg7-KO mice (Fig. 5H). To further examine FGF2 expression and localization in the kidney, we performed immunohistochemical staining (Fig. 5I). FGF2 was rarely detected in sham control kidneys. After ischemic AKI, FGF2 accumulated in the cytoplasm of atrophic tubules, primarily on the basolateral side, in WT kidneys. In contrast, this tubular expression of FGF2 was significantly suppressed in iRT-Atg7-KO mice (Fig. 5I).

### 3.5 Supplementation with glucose but not BCAAs alleviated CR-induced renal fibrosis and dysfunction following IRI

**Fig. 6:**
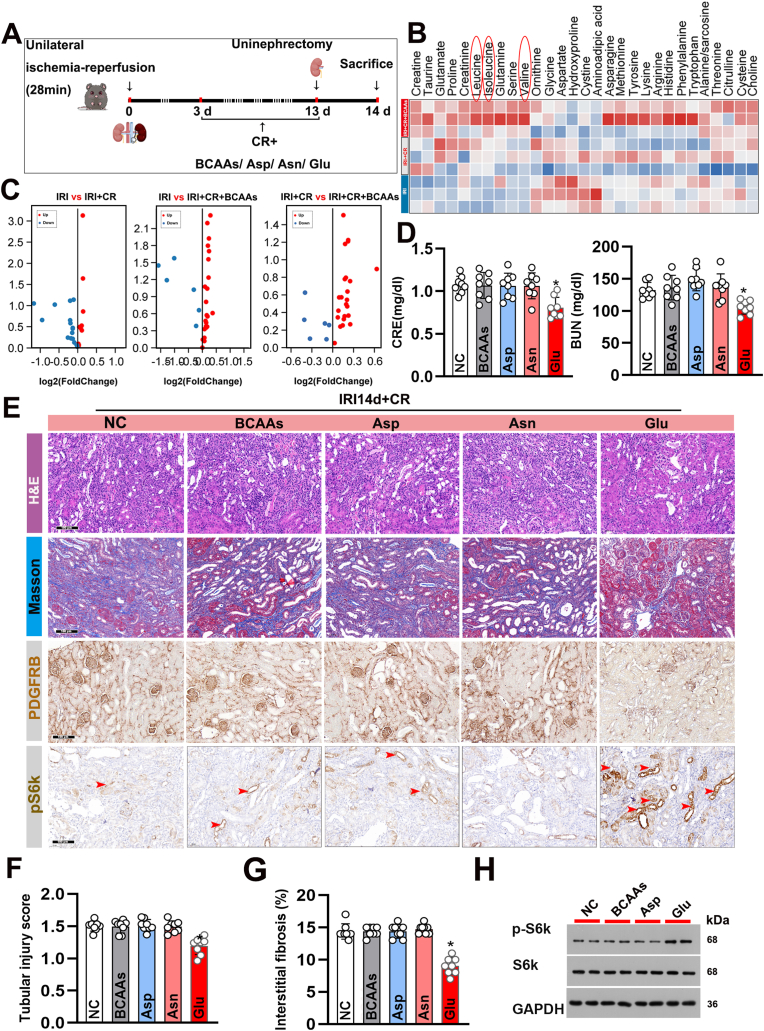
The supplementation of glucose, rather than BCAAs, can mitigate the exacerbation of CR-affected renal fibrosis and dysfunction. (A) Schematic of the experimental design. Mice underwent unilateral IRI (28 min) followed by CR with or without supplementation of BCAAs, Asp, Asn, or Glu for 14 days. (B) Heatmap showing the levels of various metabolites in the kidneys. (C) Volcano plots showing differentially expressed metabolites in the kidneys of IRI + CR mice with or without supplementation with BCAAs, Asp, Asn, or Glu. Upregulated metabolites are shown in red, and downregulated metabolites are shown in blue. (D) Serum CRE and BUN levels in the different treatment groups. Data are presented as mean ± SEM. n = 8 per group. ∗P < 0.05 versus NC. (E) Representative images of kidney sections stained with H&E, Masson's trichrome, PDGFRB, and pS6K in the different treatment groups. Red arrowheads in the pS6K panels indicate positive staining. Scale bars: 100 μm. (F) Quantification of tubular injury scores in the different groups. Data are presented as mean ± SEM. n = 8 per group. ∗P < 0.05 versus NC. (G) Quantification of interstitial fibrosis percentage in the different groups, as assessed by Masson's trichrome staining. Data are presented as mean ± SEM. n = 8 per group. ∗P < 0.05 versus NC. (H) Immunoblot analysis of pS6K, S6K, and GAPDH in kidney tissues from the different groups. Numbers represent quantification of band intensities relative to GAPDH.

mTORC1, a key regulator of nutrient sensing, plays a central role in cellular metabolism and stress responses. We investigated the effects of nutrient supplementation, including BCAAs, aspartate (Asp), asparagine (Asn), and Glu, to activate mTOR and evaluate their roles in this model of kidney injury and repair (Fig. 6A). We performed targeted metabolomics analysis to examine changes in amino acids and their derivatives in kidney tissues across different groups (Supplemental Datasets 4, 5, and 6), revealing significant differences between mice treated with CR and without CR following IRI (Fig. 6B and C). Interestingly, Glucose supplementation resulted in a significant reduction in serum CRE and BUN levels compared to the non-supplemented CR-treated mice, whereas supplementation with various amino acids did not produce similar effects (Fig. 6D). Histopathological analysis further demonstrated that only glucose supplementation led to a reduction in tubular injury and interstitial fibrosis (Fig. 6E–G). Additionally, immunohistochemical staining revealed a significant increase in pS6k expression in the glucose-supplemented group (Fig. 6E). Western blot analysis corroborated these findings, showing that glucose supplementation reactivated the mTORC1 pathway (pS6k) (Fig. 6H). These results indicate that it is the supplementation of glucose, rather than amino acids, that can alleviate the exacerbation of renal fibrosis and dysfunction induced by CR.

To elucidate this phenomenon, we performed a metabolomic analysis of kidney tissues from the model. We were surprised to find that the levels of isoleucine, leucine, and valine were unexpectedly elevated in the kidneys of mice subjected to CR, with isoleucine and leucine being particularly pronounced (SFig. 3A). This finding may explain why amino acid supplementation did not improve the adverse effects caused by CR. Further analysis of transcriptomic data from kidney tissues revealed a decrease in genes related to amino acid catabolism (Sfig. 3B) and a dysregulation in the expression of transporter-related genes (SFig. 3B). Immunofluorescence analysis further demonstrated that CR disrupted amino acid transport, as evidenced by altered expression levels of the essential amino acid transporters Slc7a8 and Slc7a5. CR appears to exacerbate abnormalities in BCAAs catabolism following IRI, leading to elevated renal BCAAs levels, indicating that the reduction in mTORC1 activity was not caused by decreased BCAAs levels.

### 3.6 Glucose is indispensable for the activation of mTORC1 in renal tubular cells

**Fig. 7:**
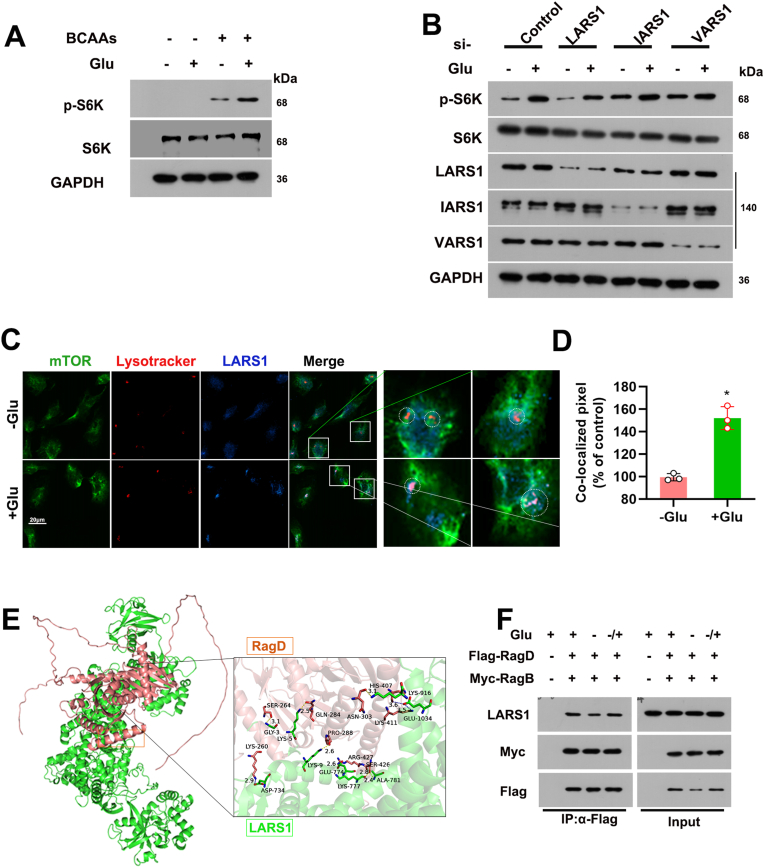
Activation of mTORC1 in renal tubular cells requires the presence of both glucose and amino acids. (A) Immunoblot analysis showing the effects of BCAAs and Glu on the p-S6K in renal tubular cells. GAPDH is shown as a loading control. (B) Western blot analysis of p-S6K, S6K, and the expression of LARS1, IARS1, and VARS1 in renal tubular cells transfected with control siRNA (si-con) or si-RNAs targeting LARS1, IARS1, or VARS1, with or without glucose. GAPDH is shown as a loading control. (C) Representative immunofluorescence images showing the colocalization of LARS1 (blue), Lysotracker (red), and mTORC1 (green) in renal tubular cells, with or without Glu. Scale bars: 20 μm. (D) The bar graph quantifies the percentage of co-localized pixels in (C) compared to the control. Data are presented as mean ± SEM. ∗P < 0.01 versus -Glu group. (E) Molecular docking was used to verify the binding activity between RagD and LARS1 proteins. The red ribbon structure represents the RagD protein, while the green ribbon structure represents the LARS1 protein. (F) Co-IP analysis showing the interaction between LARS1 and RagB/RagD in renal tubular cells, with or without glucose, in the presence of amino acids. −/+,add back Glu.

To investigate the glucose and amino acid requirements for mTORC1 activation, we assessed the effects of their supplementation on the mTORC1 signaling pathway in renal tubular cells. Western blot analysis revealed that mTORC1 activation by BCAAs was glucose-dependent (Fig. 7A). Different amino acids can activate Rag guanosine triphosphatase through their corresponding aminoacyl-tRNA synthetases, thereby activating mTORC1 [30]. By silencing the branched-chain amino acid sensors LARS1, IARS1, and VARS1, we found that LARS1 knockdown was most critical for mTORC1 activation, as its loss abrogated nutrient-induced mTORC1 signaling (Fig. 7B). In the presence of glucose and leucine, LARS1 translocates to the lysosome and facilitates the conversion of RagD-GTP to RagD-GDP, leading to mTORC1 activation [31]. Immunofluorescence confirmed the colocalization of LARS1 with mTORC1 in the presence of glucose, emphasizing the importance of glucose in mTORC1 activation (Fig. 7C and D). We downloaded 3D protein structures of LARS1 and RagD from the RCSB database (https://www.rcsb.org/). Using Discovery Studio and Ligplus software, we performed 3D and 2D force analysis and visualization (Fig. 7E). Co-IP experiments revealed an enhanced interaction between overexpressed and tagged LARS1 and RagD under glucose-rich conditions. This interaction was reduced upon glucose deprivation but was partially restored in the add-back condition (Fig. 7F). These results suggest that the interaction between LARS1 and RagD is sensitive to glucose availability.

### 3.7 ULK1 phosphorylates LARS1, inhibiting mTORC1 activation in the absence of glucose

**Fig. 8:**
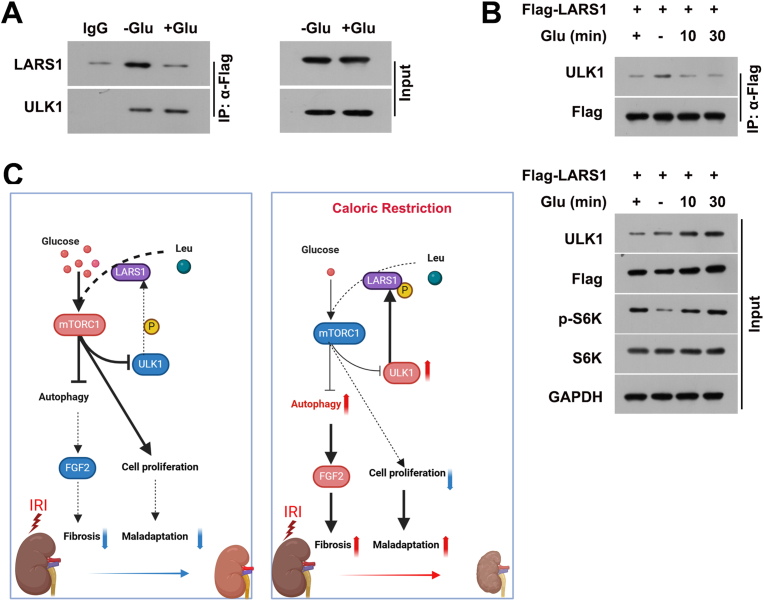
In the absence of glucose, ULK1 phosphorylates LARS1, reducing mTORC1 activation. (A) Co-IP analysis showing the interaction between LARS1 and ULK1 in renal tubular cells in the presence or absence of Glu. Western blot analysis was performed using anti-ULK1 and anti-LARS1 antibodies. IgG serves as a control (B) Western blot analysis of ULK1,p-S6K, S6K, and LARS1 in renal tubular cells transfected with Flag-LARS1, with or without Glu for the indicated times, in the presence of amino acids. GAPDH is shown as a loading control. (C) Schematic diagram of caloric restriction modulates mTORC1 signaling to influence autophagy, fibrosis, and maladaptation.

Finally, we explored the role of ULK1, a key regulator of autophagy, in modulating mTORC1 activation under nutrient-deprived conditions. LARS1 was identified as possible ULK1-interacting protein [32] and potential member of an autophagy network by mass spectrometry [33]. Co-immunoprecipitation experiments revealed that in the absence of glucose, ULK1 phosphorylates LARS1, leading to its inactivation and preventing mTORC1 activation (Fig. 8A). The association of overexpressed and tagged LARS1 and ULK1 was increased in cells deprived of glucose (Fig. 8B). These results suggest that ULK1-mediated inactivation of LARS1 underlies the failure of mTORC1 activation in CR conditions. Upon sensing leucine in the presence of glucose, LARS1 supports translation by leucylating tRNA and also by activating mTORC1. However, in the absence of glucose, LARS1 phosphorylated by ULK1 loses its leucine binding capability, decreasing both activities, thus leading to the inhibition of protein synthesis and activation of autophagy (Fig. 8C).

## 4 Discussion

In this study, we explored the impact of CR on renal injury and fibrosis following IRI, revealing that CR exacerbates renal dysfunction, fibrosis, and autophagy activation. Although CR has shown protective effects in various acute and chronic kidney diseases, these findings highlight a previously unappreciated detrimental effect of CR on renal tubule repair in the context of recovery after AKI.

The beneficial effects of CR on health are believed to stem from optimized cellular energy utilization and the induction of adaptive cellular stress responses [10]. Previous studies have shown that in the gut, fasting and refeeding can enhance intestinal stem cell function and intestinal epithelial repair [34], while in muscle, fasting induces muscle stem cells to enter a deep quiescent state, delaying muscle regeneration after injury [35]. These observations suggest that the effects of CR on various tissues and stem cell systems may be context-dependent. After kidney injury, repair may result in the formation of a disorganized cellular and extracellular matrix, or it can regenerate the original tissue structure through a regenerative process. The repair of the existing nephron units after tubular damage is an evolutionarily conserved process [36]. CR triggers evolutionarily conserved adaptive responses, halting renal tissue repair to conserve resources and attempting to promote adaptation and survival, with mTOR playing a key role in this process, as revealed in this study.

mTORC1, a central regulator of cell growth and metabolism, integrates signals from nutrients, energy status, and stress [37]. However, the effects of mTORC1 inhibition can be context-dependent, with studies indicating both protective and potentially harmful effects in different disease models. For example, recent research has shown that CR during pregnancy affects mTORC1 signaling, reducing nephron progenitor cell proliferation in offspring, which may impair kidney function later in life [38]. Notably, supplementation with methionine or increasing mTORC1 activity during pregnancy reversed the negative effects of CR on nephron progenitor cell proliferation. Our results demonstrate that CR and mTORC1 inhibition significantly worsens renal outcomes after IRI, as evidenced by renal function impairment, and exacerbated tubular injury and interstitial fibrosis. mTORC1 is activated by growth factors, nutrients, and cellular energy status, while under energy-deficient conditions, its activity is downregulated to limit cellular growth. In the injured kidney, mTORC1 activation under nutrient-rich conditions promotes tubular cell proliferation and recovery [14]. However, under nutrient-deficient conditions, the injured kidney becomes a victim of the systemic downregulation of mTORC1 activity, leading to impaired renal repair and exacerbated fibrosis. Forced overexpression of S6K1 in the injured kidney to activate the mTORC1-S6K1 downstream pathway could potentially balance systemic nutrient supply, rescue the damaged kidney, and mitigate the detrimental effects associated with CR.

Rapamycin with immunosuppressive and antitumor effects, is widely used in combination with calcineurin inhibitors to enhance immunosuppression while mitigating their side effects [39]. IRI is a major cause of DGF [23], and the use of rapamycin in DGF patients provides a valuable clinical model to observe the effects of mTOR inhibition on renal repair. A comprehensive literature search was conducted using PubMed, Embase, Scopus, Cochrane Library, Web of Science, Ovid, and ProQuest, identifying five studies [[40], [41], [42], [43], [44]] that reported the effect of rapamycin on the duration of DGF in patients after renal transplantation. These studies demonstrated longer DGF durations in rapamycin-treated groups compared to controls, although not all studies reported statistically significant differences. Specifically, in Kelly D. Smith et al. reported the mean DGF duration was 27.6 days with rapamycin versus 6.4 days without rapamycin [41]. Laetitia Albano et al. demonstrated that the values were 10.2 days and 7.6 days, respectively [44]. Similarly, Ryan A. McTaggart et al. reported a mean duration of 14.86 days in the rapamycin group versus 9.59 days in the control group [42]. Stallone Giovanni et al. showed the DGF duration was 6.2 days with rapamycin and 3.2 days without rapamycin [40]. Finally, W. Tahira 2015 observed a substantial increase in DGF duration in the rapamycin group (42.3 days) compared to the control group (26.5 days) [43]. This suggests that the mechanism by which mTOR inhibition impedes post-ischemic kidney repair may be a common feature in humans.

CR inhibits mTORC1 activity, thereby reducing cellular growth signals and activating autophagy—a process that is generally protective by clearing damaged organelles and proteins [19]. The link between CR, autophagy, and mTORC1 inhibition provides a potential mechanistic explanation for the observed renal damage and renal fibrosis. We demonstrated that the autophagy inhibitor 3-MA and Atg7 gene knockout mitigated CR-induced renal injury, suggesting that the exacerbation of renal damage by CR was at least partially mediated through autophagy. This finding aligns with previous studies showing that dysregulated autophagy can contribute to the progression of renal fibrosis and tubular injury by paracrine secretion of pro-fibrotic cytokines [45]. By inhibiting autophagy, tubular Atg7 ablation reduced fibrosis by decreasing the pro-fibrotic factor FGF2, supporting the hypothesis that excessive autophagy plays a crucial role in caloric restriction-induced renal damage. Interestingly, rapamycin mimicked the effects of CR on renal injury and autophagy activation, further corroborating the involvement of the mTORC1-autophagy axis in mediating the effects of CR. This highlights the complexity of mTORC1 signaling in kidney disease, where its inhibition can have both protective and detrimental effects depending on the context and extent of autophagy activation.

Given that BCAAs are critical for mTORC1 activation, their depletion likely contributes to the impaired mTORC1 signaling observed in CR-treated mice. Supplementation with BCAAs may potentially benefit kidney recovery. Interestingly, targeted metabolomics detected an increase, rather than a decrease, in BCAAs. This may reflect a reduction in amino acid catabolism, enhanced absorption capacity, and the production of amino acids via cellular autophagy. This also explains why exogenous supplementation of BCAAs indeed increased the levels of BCAAs in the kidneys, but did not benefit kidney repair. We noticed that CR significantly reduced fasting blood glucose, so the inhibition of mTORC1 may be attributed to the decrease in blood glucose. Interestingly, glucose supplementation improved fibrosis and reduced excessive autophagy in the kidneys. This finding is consistent with the notion that both glucose and amino acid availability are essential for mTORC1 activity [46], and their disruption can have significant consequences for cellular function and survival [47]. Finally, we explored the mechanism by which glucose regulates mTORC1. Our study also identified a key role for ULK1, a regulator of autophagy, in modulating LARS1 activity and mTORC1 signaling. We found that in the absence of glucose, ULK1 phosphorylates LARS1, leading to its inactivation and subsequent failure to activate mTORC1. This mechanism likely contributes to the inability of mTORC1 to be activated under CR conditions, further exacerbating renal injury. The interplay between nutrient availability, ULK1 activity, and mTORC1 signaling underscores the complexity of metabolic regulation in the kidney and its impact on disease outcomes.

Overall, our findings suggest that CR application following IRI, may be detrimental due to its effects on mTORC1 signaling and autophagy. The potential therapeutic implications of targeting the mTORC1-autophagy axis in kidney disease warrant further investigation, particularly in the condition of tissue regeneration, such as recovery after AKI. Future studies should explore the long-term effects of CR in different models of kidney injury and identify potential strategies to modulate autophagy and mTORC1 signaling to optimize renal outcomes.

## CRediT authorship contribution statement

**Lang Shi:** Writing – review & editing, Writing – original draft, Software, Methodology, Formal analysis, Data curation, Conceptualization. **Hongchu Zha:** Methodology, Formal analysis, Data curation. **Juan Zhao:** Methodology, Formal analysis. **Haiqian An:** Methodology. **Hua Huang:** Methodology. **Yao Xia:** Methodology. **Ziyu Yan:** Methodology. **Zhixia Song:** Methodology. **Jiefu Zhu:** Writing – review & editing, Writing – original draft, Validation, Supervision, Resources, Project administration, Investigation, Conceptualization.

## Statement of ethics

All animal care and experimental programs are in the compliance with animal management regulations of the Ministry of Health of the People's Republic of China and approved by the Ethics Committee of the Three Gorges University (202205010T2).

## Declaration of competing interest

The authors have no conflicts of interest to declare.

## Data Availability

No data was used for the research described in the article.

## Appendix A Supplementary data

Supplementary Figure 1. Overexpression of S6K activates mTORC1 and mitigates CR-induced renal fibrosis and dysfunction following IRI.

Supplementary Figure 2. The autophagy inhibitor 3-MA alleviates CR-induced renal injury and excessive autophagy activation following IRI.

Supplementary Figure 3. CR exacerbates branched-chain amino acid metabolism abnormalities after IRI, leading to elevated BCAAs levels.

Supplementary Figure 4. Postoperative changes in body weight, blood glucose levels, and kidney injury markers in mice.

Supplemental Datasets 1. The RNA sequencing data for the comparison between IRI-14d and IRI-14d + CR groups.

Supplemental Datasets 2. The RNA sequencing data for the comparison between IRI-14d + Rap and IRI-14d + CR groups.

Supplemental Datasets 3. The RNA sequencing data for the comparison between IRI-14d + Rap and IRI-14d groups.

Supplemental Datasets 4. Targeted metabolomics (amino acids and their derivatives) data for comparing the IRI + CR group and IRI group.

Supplemental Datasets 5. Targeted metabolomics (amino acids and their derivatives) data for comparing the IRI + CR + BCAAs group and IRI group.

Supplemental Datasets 6. Targeted metabolomics (amino acids and their derivatives) data for comparing the IRI + CR + BCAAs group and IRI + CR group.
