# Supplementary material for: Caloric restriction exacerbates renal post-ischemic injury and fibrosis by modulating mTORC1 signaling and autophagy
Source: Redox Biol. 2025 Jan 16;80:103500. doi: 10.1016/j.redox.2025.103500 (PMC11787690; doi:10.1016/j.redox.2025.103500)
Supplement: Multimedia component 7 [file mmc7.docx]

**Caloric Restriction Exacerbates Renal Injury and Fibrosis Following Ischemia-Reperfusion by Modulating mTORC1 Signaling and Autophagy**

Lang Shi^1,2^, Hongchu Zha^3^, Juan Zhao^4^, Haiqian An^1,2^, Hua Huang^3^, Yao Xia^3^, Ziyu Yan^3^, Zhixia Song ^5^, Jiefu Zhu ^6*^

1 Department of Nephrology, The First Hospital of Lanzhou University, Lanzhou 730000, China.

2 The First Clinical Medical College, Lanzhou University, Lanzhou 730000, China.

3 Department of Nephrology, The First Clinical Medical College of Three Gorges University, Center People’s Hospital of Yichang, Yichang 443000, China.

4 Department of Laboratory Medicine, The First Hospital of Lanzhou University, Lanzhou 730000, China.

5 Department of Nephrology, The People's Hospital of Longhua, Shenzhen 518109, China.

6 Department of Organ Transplantation, Renmin Hospital of Wuhan University, Wuhan 430060, China.

^*^ Corresponding author. E-mail address: Jiefu Zhu: [jiefuzhu@whu.edu.cn](mailto:jiefuzhu@whu.edu.cn)

**Supplementary Figures**

**Supplementary Figure 1.**

| 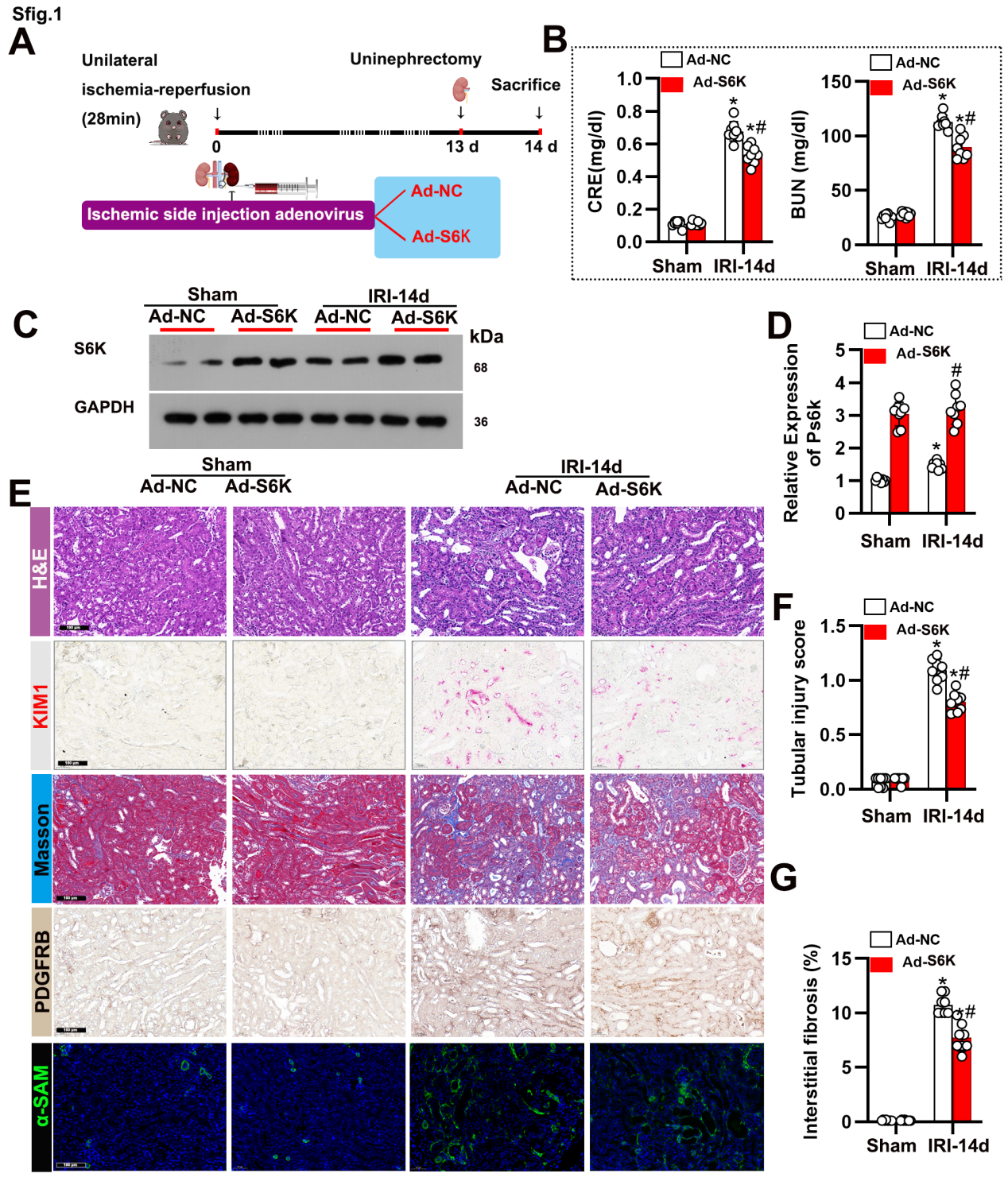 |
| --- |
| **Supplementary Figure 1. Overexpression of S6K activates mTORC1 and mitigates CR-induced renal fibrosis and dysfunction following IRI.**  (A) Schematic of the experimental design. Mice underwent unilateral IRI (28 minutes) followed by uninephrectomy and injection of adenovirus encoding either a Ad-NC or Ad-S6K into the ischemic kidney. (B) Serum CRE and BUN levels in sham and IRI-14d mice treated with Ad-NC or Ad-S6k. Data are presented as mean ± SEM. n = 8 per group. *P < 0.05 versus Sham; #P < 0.05 versus Ad-NC. (C) Western blot analysis of S6K expression in kidney tissues from sham and IRI-14d mice treated with Ad-NC or Ad-S6k. GAPDH was used as a loading control. (D) Quantification of S6K expression levels from Western blots. Data are presented as mean ± SEM. n = 8 per group. *P < 0.05 versus Sham; #P < 0.05 versus Ad-NC. (E) Representative images of kidney sections stained with H&E, KIM1, Masson’s trichrome, PDGFRB, and α-SMA in sham and IRI-14d mice treated with Ad-NC or Ad-S6K. Scale bars: 100 μm. (F) Quantification of tubular injury scores in the different groups. Data are presented as mean ± SEM. n = 8 per group. *P < 0.05 versus Sham; #P < 0.05 versus Ad-NC. (G) Quantification of interstitial fibrosis percentage in the different groups, as assessed by Masson’s trichrome staining. Data are presented as mean ± SEM. n = 8 per group. *P < 0.05 versus Sham; #P < 0.05 versus Ad-NC. |

**Supplementary Figure 2.**

| 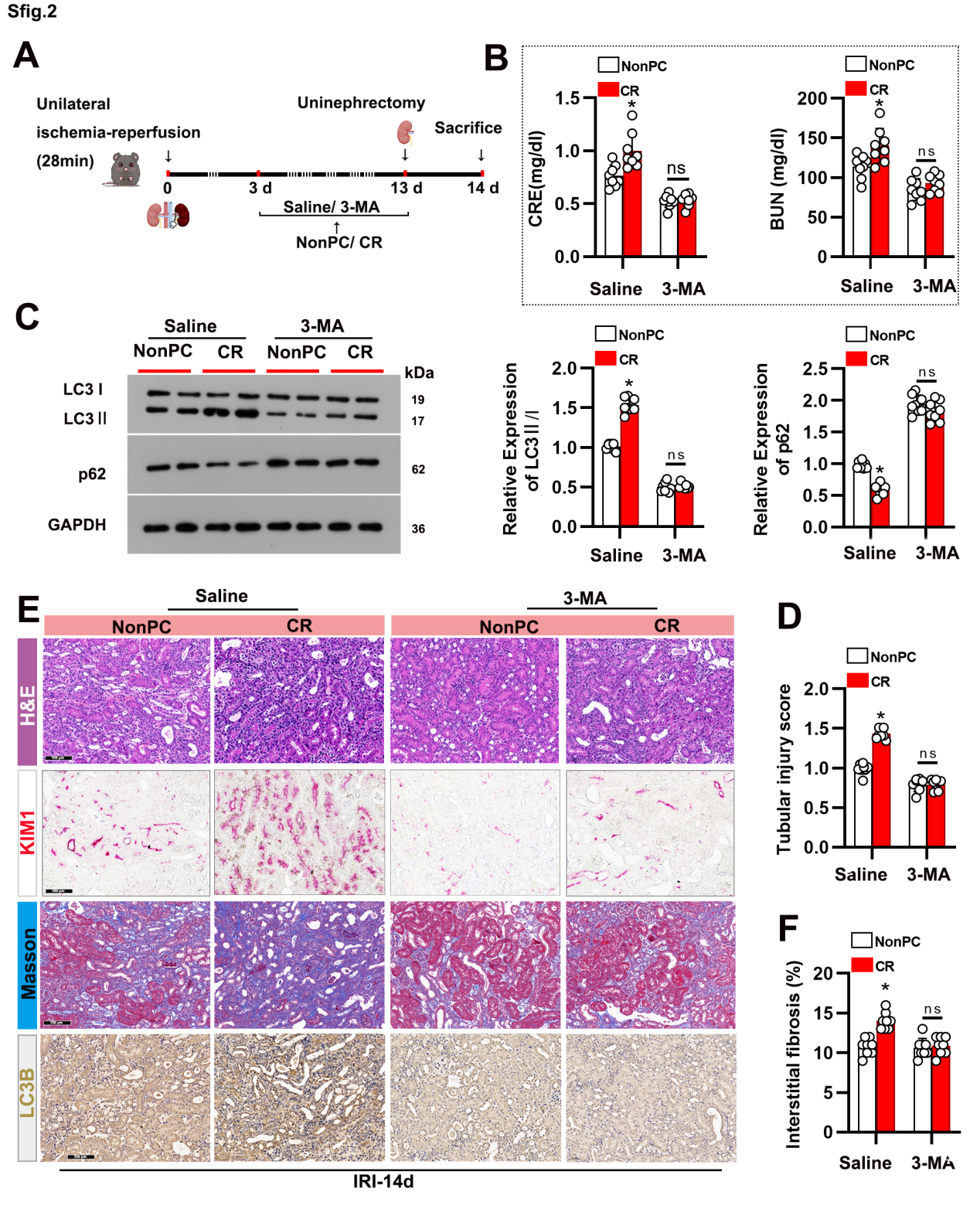 |
| --- |
| **Supplementary Figure 2. The autophagy inhibitor 3-MA alleviates CR-induced renal injury and excessive autophagy activation following IRI.**  (A) Schematic of the experimental design. Mice underwent unilateral IRI (28 minutes) followed by uninephrectomy and treatment with saline or 3-MA, with or without CR, for 14 days. (B) Serum CRE and BUN levels in IRI-14d mice treated with saline or 3-MA under NonPC and CR conditions. Data are presented as mean ± SEM. n = 8 per group. *P < 0.05 versus Saline NonPC; ns, not significant. (C) Western blot analysis of LC3B, and p62 in kidney tissues from IRI-14d mice treated with saline or 3-MA under NonPC and CR conditions. GAPDH was used as a loading control. Numbers represent quantification of band intensities relative to GAPDH. (D) Quantification of tubular injury scores in IRI-14d mice treated with saline or 3-MA under NonPC and CR conditions. Data are presented as mean ± SEM. n = 8 per group. *P < 0.05 versus Saline NonPC; ns, not significant. (E) Representative images of kidney sections stained with H&E, KIM1, Masson’s trichrome, and LC3 in IRI-14d mice treated with saline or 3-MA under NonPC and CR conditions. Scale bars: 100 μm. (F) Quantification of interstitial fibrosis percentage in the different groups, as assessed by Masson’s trichrome staining. Data are presented as mean ± SEM. n = 8 per group. *P < 0.05 versus Saline NonPC; ns, not significant. |

**Supplementary Figure 3.**

| 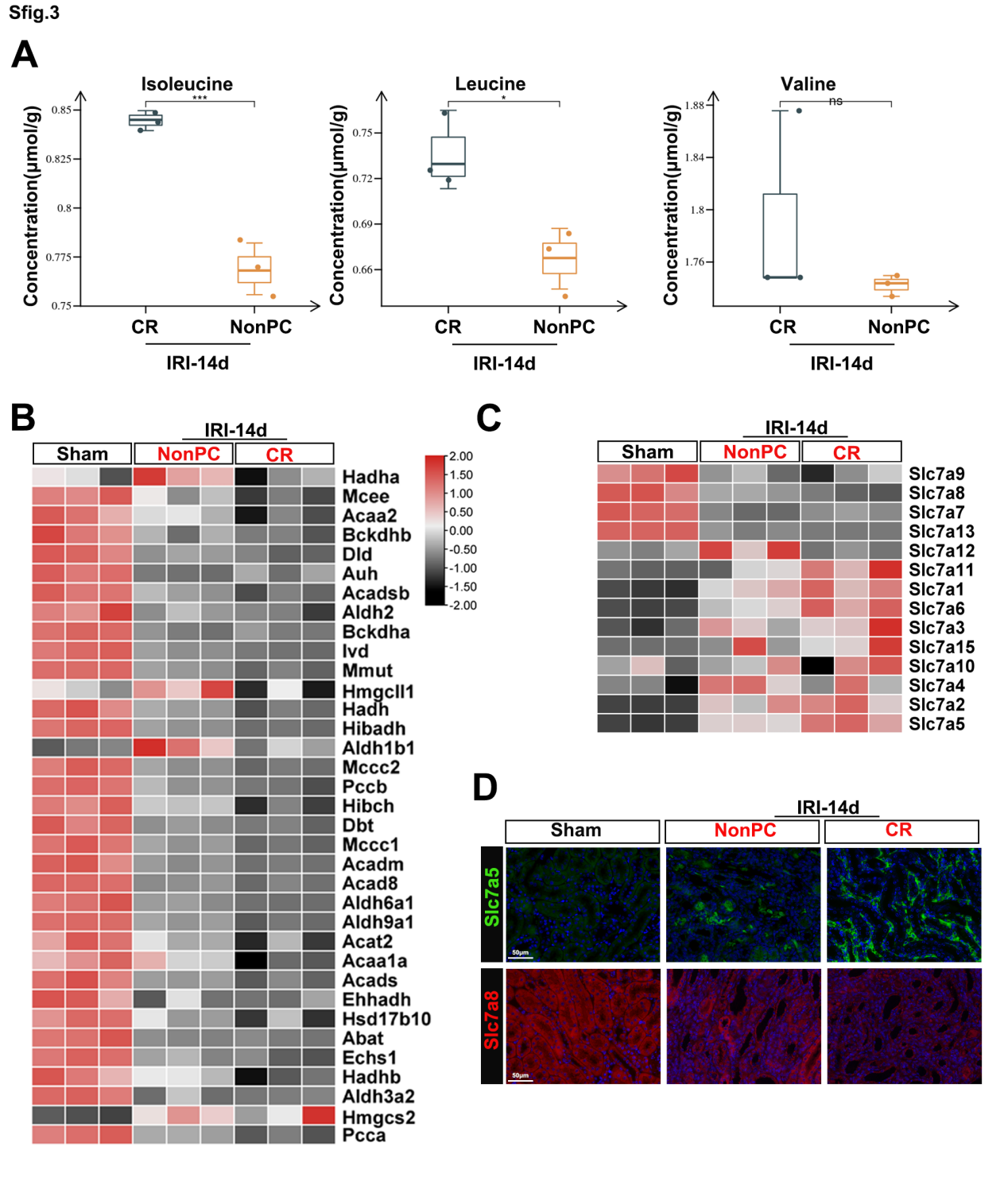 |
| --- |
| **Supplementary Figure 3. CR exacerbates branched-chain amino acid metabolism abnormalities after IRI, leading to elevated BCAAs levels.**  (A) Box plots showing the concentrations of isoleucine, leucine and valine in the kidneys of IRI-14d mice under NonPC and CR conditions. Data are presented as mean ± SEM. n = 3 per group. *P < 0.05, **P < 0.001. ns, not significant. (B) Heatmap showing the expression levels of genes involved in BCAAs metabolism in the kidneys of Sham, IRI-14d mice under NonPC and CR conditions. Data are normalized to the mean expression level of each gene. (C) Heatmap of BCAAs transporter gene expression levels in the kidneys of Sham and IRI-14d mice under NonPC and CR conditions. Data are normalized to the mean expression level of each gene. (D)Representative images of kidney sections stained with Slc7a5 and Slc7a8 in IRI-14d mice under NonPC and CR conditions. Scale bars: 50 μm. |

**Supplementary Figure 4.**

| 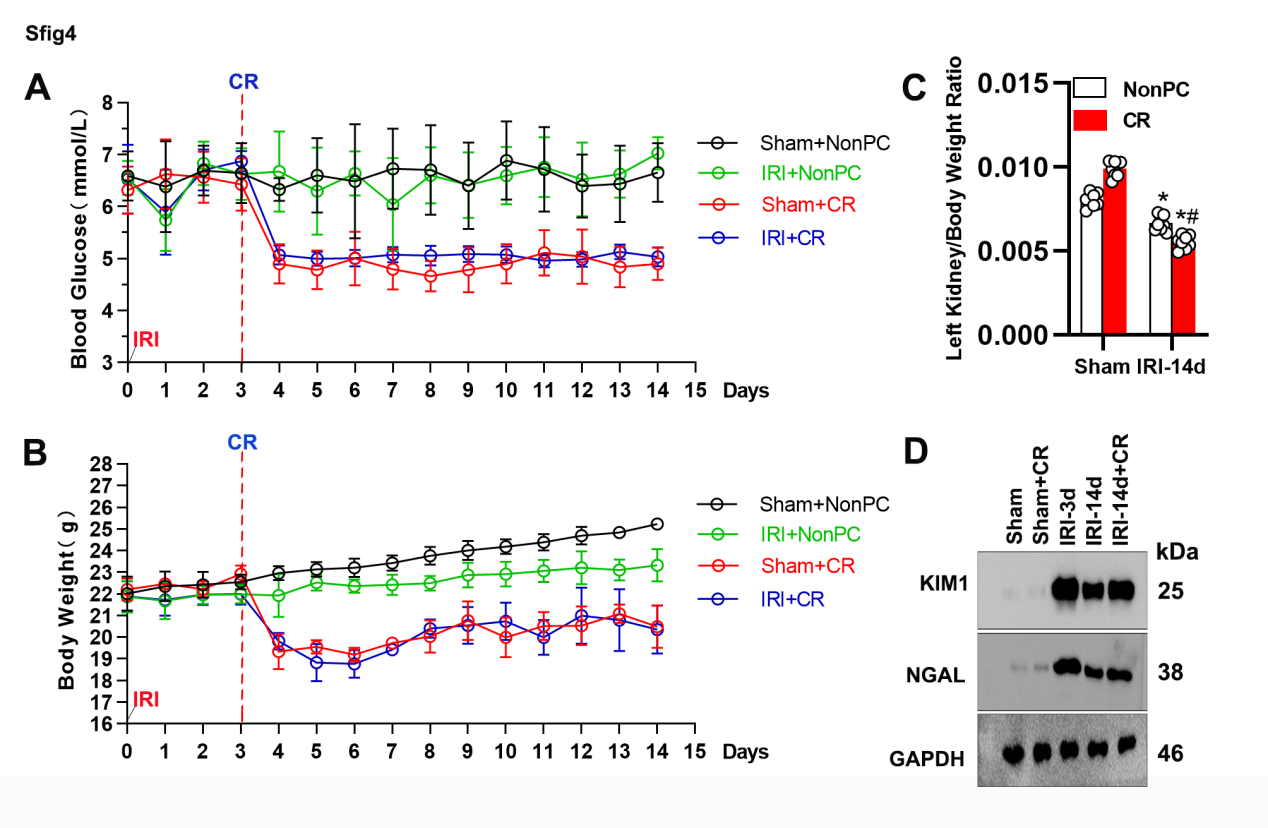 |
| --- |
| **Supplementary Figure 4. Postoperative changes in body weight, blood glucose levels, and kidney injury markers in mice.**  (A) Changes in blood glucose levels. (B) Changes in body weight. (C) Kidney-to-body weight ratio. (D) Changes in injury markers KIM1 and NGAL.*P < 0.05 versus Sham; #P < 0.05 versus NonPC. |
